# How Feasible is Extracorporeal Cardiopulmonary Resuscitation in a Medium Urban Population Centre?

**DOI:** 10.7759/cureus.6324

**Published:** 2019-12-08

**Authors:** Derek Rollo, Paul Atkinson, Jay Mekwan, Sohrab Lutchmedial, Joanna Middleton, James French, Steve Chanyi, James Gould, George Kovacs, Jean-François Légaré, Mark Tutschka, Jacqueline Fraser, Michael Howlett

**Affiliations:** 1 Family Medicine, Saint John Regional Hospital, Saint John, CAN; 2 Emergency Medicine, Saint John Regional Hospital, Saint John, CAN; 3 Emergency Medicine, Horizon Health Network, Saint John, CAN; 4 Cardiology, New Brunswick Heart Centre, Saint John Regional Hospital / Dalhousie University, Saint John, CAN; 5 Cardiac/Thoracic/Vascular Surgery, Saint John Regional Hospital, Saint John, CAN; 6 Emergency Medicine, Queen Elizabeth II Health Science Center / Dalhousie University, Halifax, CAN; 7 Emergency Medicine, Dalhousie University, Halifax, CAN; 8 Cardiac Surgery, Saint John Regional Hospital / Dalhousie University, Saint John, CAN; 9 Critical Care Medicine, Saint John Regional Hospital / Dalhousie University, Saint John, CAN

**Keywords:** cardiac arrest, resuscitation, extracorporeal membranous oxygenation

## Abstract

Background

Patients suffering from out-of-hospital cardiac arrest (OHCA) experience poor survival and neurological outcomes, with rates remaining relatively unchanged despite advancements. Extracorporeal membrane oxygenation (ECMO), termed extracorporeal cardiopulmonary resuscitation (ECPR) in arrests, may offer improved outcomes. We developed local screening criteria for ECPR and then estimated the frequency of use by applying those criteria retrospectively to a cardiac arrest database. The purpose was to determine if an ECPR program is feasible in a medium urban population centre in Atlantic Canada.

Methods

A three-round modified Delphi survey, building upon data from a literature review, was conducted in collaboration with external experts. The resulting selection criteria for potential ECPR candidates were applied to a pre-existing local cardiac arrest database, supplemented by health records review, identifying potential candidates eligible for ECPR.

Results

Consensus inclusion criteria included witnessed cardiac arrest, age <70, refractory arrest, no-flow time <10min, total downtime <60min, and presumed cardiac or selected non-cardiac etiologies. Consensus exclusion criteria were an unwitnessed arrest, asystole, and select etiologies and comorbidities. Simplified criteria were developed to facilitate emergency medical services transport. Historically, 20.0% (95% CI 16.2-24.3%) of OHCA would be transported to the Emergency Department (ED), with 4.9% (95% CI 3.0% to 7.6%) qualifying for ECPR.

Conclusion

Despite conservative estimates based upon historically small numbers of select cardiac arrest patients meeting eligibility for transport and initiation of ECPR, a dedicated program may be feasible in our regional hospital setting. Patient care volumes suggest it would not be resource intensive yet would be sufficiently busy to maintain competency.

## Introduction

Rates of neurologically intact survival from out of hospital cardiac arrest (OHCA) in adult patients are poor, with poor long-term outcomes [1]. The use of veno-arterial Extracorporeal Membrane Oxygenation (ECMO) in cardiac arrest, termed extracorporeal cardiopulmonary resuscitation (ECPR), may maintain vital organ perfusion, buying time for investigation and treatment of reversible causes of refractory arrest. ECPR is recognized internationally more recently as a potential tool to assist resuscitative efforts in the emergency department (ED) during cardiac arrest [2]. Observational data is suggestive that refractory OHCA treated with ECPR may lead to improved outcomes over those treated with conventional resuscitation, with survival rates as high as 48% [3].

Tertiary healthcare facilities based in smaller and medium urban centres that provide interventional cardiac services face the uncertainty of how feasible and sustainable the introduction of an ECPR program is in their setting. We developed site-specific screening criteria for ECPR to predict the frequency of local ECPR events and applied those criteria retrospectively to a cardiac arrest database. Results were used to determine the feasibility of an ECPR program in our small metropolitan area within Atlantic Canada.

## Materials and methods

This study was undertaken using a sequential modified Delphi and database review methodology.

Determination of local ECPR selection criteria

Published patient selection criteria currently in use for ECPR were identified by literature review [4-9]. This list formed the basis of the modified Delphi survey, involving 13 local, regional, national, and international experts, including members representing pre-hospital care, emergency medicine, cardiology, intensive care medicine, cardiac surgery, anesthesia, and perfusion technology. The team liaised with established ECPR programs in Vancouver, British Columbia, and Sydney, New South Wales. Proposed transport and team activation inclusion and exclusion criteria were scored by a panel of 13 experts. A threshold of 60% consensus was required to progress to the next round, with a final consensus rate of over 80% required to close the survey. Additional local logistical factors were also reviewed. The final New Brunswick (NB) ECPR criteria were agreed upon following three rounds and additional review by an international expert.

Estimation of ECPR frequency

A cardiac arrest database review, supplemented by a health records review for missing data, was performed following the REporting of studies Conducted using Observational Routinely-collected Data (RECORD) guidelines.

The NB-ECPR criteria were applied retrospectively to the complete database of adult patients presenting to an ED serving a medium urban population centre, in cardiac arrest, over a five-year period. Data were analyzed using standard parametric and non-parametric measures. Confidence intervals (CI) for proportions of 95% were calculated using the modified Wald method using GraphPad Software QuickCalcs, GraphPad Software, La Jolla California USA, https://www.graphpad.com/quickcalcs/confInterval1/. 

## Results

Modified Delphi survey

First-round responses achieved ≥87.5% consensus for the selection of exclusion criteria. Inclusion criteria had agreement ≥62.5%. Responses to the second round for inclusion criteria were unanimous at 100% with the exception of age parameters (<65 years versus <70 years). Age was proposed to be <70 years following ECPR team consultation. The final set of NB-ECPR criteria for transport to hospital, and for ECPR team activation (Table 1) achieved 100% consensus though subsequent expert review refined additional exclusion criterion based on experience (asystole). Inclusion criteria were witnessed cardiac arrest, age <70, refractory arrest, no-flow time <10 minutes, total downtime <60 minutes, and presumed cardiac or selected non-cardiac etiologies. Exclusion criteria were unwitnessed arrest, asystole, select etiologies, and comorbidities. Simplified criteria were developed to facilitate emergency medical services (EMS) transportation.

**Table 1:** Final NB-ECPR criteria for transport to hospital and for ECPR team activation NB, New Brunswick; ECPR, extracorporeal cardiopulmonary resuscitation; DNR, do not resuscitate; ADL, activities of daily living; OD, overdose

Criteria for Transport To Hospital		Criteria for ECPR Team Activation	
Inclusion Criteria	Exclusion Criteria	Inclusion Criteria	Exclusion Criteria
	✗ Unwitnessed Cardiac Arrest	✓ Witnessed Cardiac Arrest	✗ Unwitnessed Cardiac Arrest
	✗ Initial Rhythm Asystole	✓ Age <70 Years Old	✗ Asystole at Scene
✓ Witnessed Cardiac Arrest	✗ Suspected Etiology:	✓ No Flow Time <10min	✗ Suspected Etiology:
	-Uncontrolled Hemorrhage	✓ Total Downtime <60min	-Uncontrolled Hemorrhage
✓ Age <70 years old	-Irreversible Brain Damage	✓ Refractory Cardiac Arrest	-Irreversible Brain Damage
	-Trauma	✓ Suspected Etiology:	-Trauma
✓ No Flow Time <10 minutes	✗ Comorbidity:	-Cardiac	✗ Comorbidity:
	-Severe Disability Limiting ADLs	-Pulmonary Embolism	-Severe Disability Limiting ADLs
	-Standing DNR Order	-Drug OD / Poisoning	-Standing DNR Order
	-Undergoing Palliation	-Hypothermia	-Undergoing Palliation

Expected frequency of ECPR candidates

Complete data were available for 273 patients presenting to the ED in cardiac arrest (see Appendix and Tables 2-3 for candidate breakdown).

**Table 2:** Absolute numbers of candidates meeting transport criteria EMS, emergency medical services; ADL, activities of daily living; DNR, do not resuscitate; ADL, activities of daily living

Inclusion Criteria	Count (n)
Witnessed Cardiac Arrest	186
Age <70yo	173
No Flow <10min	146
Full Inclusion Criteria	82
Exclusion Criteria	
Unwitnessed Cardiac Arrest	87
“Asystole” at Scene	160
Suspected Etiology:	
-Uncontrolled Bleed	No Data
-Irreversible Brain Damage	No Data
-Trauma	10
Comorbidity:	
-Disability Limiting ADL	No Data
-Standing DNR Order	0
-Undergoing Palliation	6
Total Excluded ≥1 Criteria	101
-With EMS Rhythm	198

**Table 3:** Absolute numbers of candidates meeting ECPR team activation criteria ED, emergency department; ADL, activities of daily living; DNR, do not resuscitate

Inclusion Criteria	Count (n)
Witnessed Cardiac Arrest	186
Age <70 years old	173
No Flow <10min	146
Total Downtime <60 min	134
Refractory Arrest	257
Suspected Etiology:	
-Cardiac	No Data
-Select Non-Cardiac	No Data
Full Inclusion Criteria	80
-With Downtime	58
Exclusion Criteria	
Unwitnessed Cardiac Arrest	87
Asystole in ED	189
Suspected Etiology:	
-Uncontrolled Bleed	No Data
-Irreversible Brain Damage	No Data
-Trauma	10
Comorbidity:	
-Disability Limiting ADL	No Data
-Standing DNR Order	0
-Undergoing Palliation	6
Total Excluded ≥1 Criteria	211

In all, 19 patients per year or 20.0% (95% CI 16.2-24.3%) of OHCA patients met eligibility for transportation to the ED. If an EMS rhythm criteria for asystole was included, only 10% (95% CI 7.3-13.5%) would qualify. In the ED, five patients per year, or 4.9% (95% CI 3.0-7.6%) would be eligible to receive ECPR. If local, in-house cardiac catheterization hours limitations are applied, then 9.4% (95% CI 6.8-12.9%) would be eligible for transport from the field. For ED ECPR activation, 3.0% (95% CI 1.6-5.3%) would be eligible. Further details are provided in Tables 4-5.

**Table 4:** Eligible candidates for transport by EMS EMS, emergency medical services

Eligible Candidates For Transport	Total Database (%)	Yearly count (mean)
Without EMS Rhythm Criteria	20.0 [95%CI 16.2-24.3]	18.5
With EMS Rhythm Criteria	10.0 [95%CI 7.3-13.5%]	9.2
Considering In-House Cath Lab Hours		
Without Rhythm	9.4 [95%CI 6.8-12.9]	8.7
With Rhythm	5.4 [95%CI 3.5-8.2]	5

**Table 5:** Eligible candidates for ECPR ECPR, extracorporeal cardiopulmonary resuscitation

Eligible Candidates For ECPR	Total Database (%)	Yearly Count (mean)
Without Downtime Criteria	4.9 [95%CI 3.0-7.6]	4.5
With Downtime Criteria	3.8 [95%CI 2.2-6.3]	3.5
Considering In-House Cath Lab Hours		
Without Downtime	3.0 [95%CI 1.6-5.3]	2.7
With Downtime	2.4 [95%CI 1.2-4.6]	2.3

## Discussion

Despite the growing literature on ECPR practices internationally, there are no proven optimized selection criteria, and certainly none for small or medium urban centres. In this study, we have developed consensus-based criteria for ECPR and have demonstrated that the expected frequency is reasonable both in terms of skills maintenance and resource use.

Miscellaneous topics essential for the execution of a novel local ECPR program were also addressed. Mechanical CPR is an effective method for providing CPR to cardiac arrest patients and there has been evidence to show that CPR quality is greatly improved in transport while using a mechanical device [10]. Therefore, as shown in our modified Delphi, mechanical CPR devices will have a role in any future ECPR programs for OHCA. Other skills that were identified that need to be developed or sourced include rapid canalization of large blood vessels. Our Delphi showed that point-of-care ultrasound would be required for an ED-based ECPR program for vascular access and line placement.

Eligible candidates were analyzed for transport to ED by EMS, with and without the use of the EMS rhythm criterion of asystole, as historically rhythm was often categorized simply as shockable or non-shockable in the EMS data, reducing expected frequencies.

For ECPR team availability, it was agreed that initially, the program should be offered while cardiac catheterization laboratory staff are in-house. This reduces potential complicating factors of utilizing on-call resources, which can create logistical barriers and a reduction in efficiency, leading to delays. This further reduces expected candidate frequencies and hours of operation will be reevaluated during program development with the ultimate goal of unimpeded access to this service.

Finally, ECPR was never intended to be a long-term life support technique, but rather a method to extend resuscitation safely, allowing time for identifying and treating reversible causes of cardiac arrest. As such, we determined that rewarming and ECMO withdrawal should begin 24 hours post-admission, which is consistent with the existing literature [11].

Limitations

The inherent limitations of using expert opinion as a reference standard for the survey and a retrospective health records and database review are acknowledged. A contemporary literature review and liaison with international experts in the field not involved in the survey was an attempt to mitigate this. In addition, the cardiac arrest database was not designed specifically to answer this research question.

Certain conservative assumptions were applied when interpreting data. For example, patients with no cardiac arrest start time, that were witnessed arrests, were assumed to have an arrest at a time in keeping with the time of the 911 call. Total downtime was calculated using the arrival time compared with the cardiac arrest start time. Two particular limitations of the database were accounted for in the analysis. During the timeframe data was collected, all EMS in NB had automated external defibrillators, only showing shockable or non-shockable rhythms. Thus, all non-shockable rhythms were assumed asystole for secondary analysis, even though pulseless electrical activity (PEA) would be an acceptable rhythm.

In addition, we did not include patients who were not transported to hospital by EMS. Application of selection criteria to that group might impact transfer rates and subsequently impact our estimate of frequency for ECMO use.

## Conclusions

Application of expert consensus-derived selection criteria for ECPR in a medium urban population centre indicates that up to one-fifth of adult out-of-hospital cardiac arrest patients would be eligible for transport to the ED for consideration of ECPR. A smaller proportion would meet the criteria for initiating ECMO in the ED. This suggests that an NB-ECPR program would not be resource-intensive yet would be sufficiently busy to maintain adequate team competency.

## Figures and Tables

**Figure 1:**
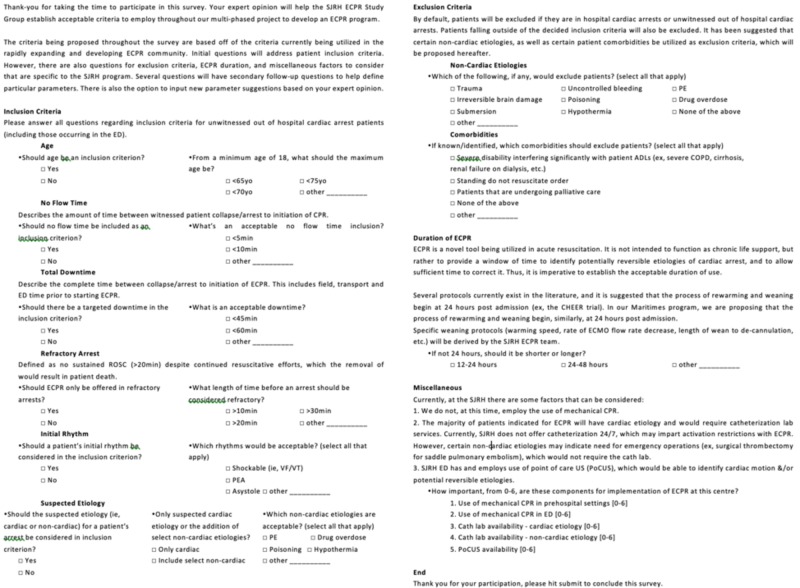
Survey 1

**Figure 2:**
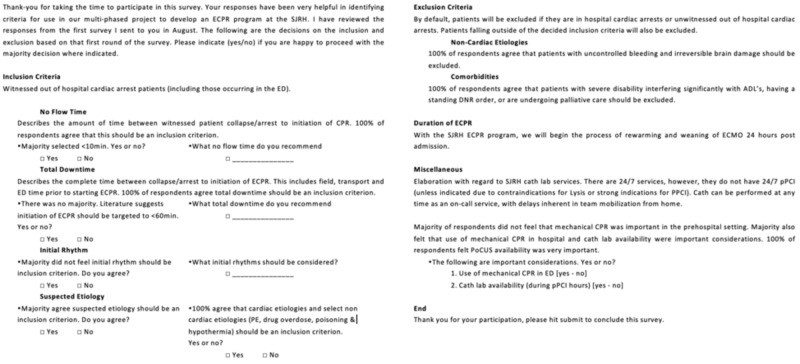
Survey 2

**Figure 3:**
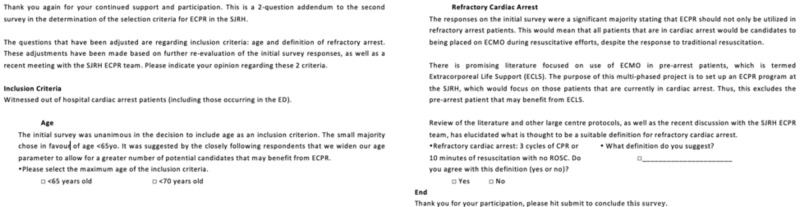
Addendum to Survey 2

## Appendices

1. New Brunswick Extracorporeal Cardiopulmonary Resuscitation (NB-ECPR) Criteria Selection Survey Number 1

2. New Brunswick Extracorporeal Cardiopulmonary Resuscitation (NB-ECPR) Criteria Selection Survey Number 2

3. Description of data extraction process

Chart review for patients in the database with missing information included patient age, pre-arrest DNR status, terminal illness status, witnessed arrest, trauma, EMS rhythm, ED rhythm, cardiac arrest start time, time of first CPR, arrival time to hospital, and survival to admission.

After chart review, the database encompassed 395 patients. Upon review, patients who did not lose a pulse, and as such did not suffer true cardiac arrest, were excluded, leaving 371 potential candidates. Further, those with missing data points beyond the chart review were excluded from the application of criteria. There were 15 with lost charts, 15 under the age of 18, 16 with a DNR, 44 because of pre-ED return of spontaneous circulation (ROSC), 3 that had ED arrests without data, 3 that were completely blank, and 2 for unknown reasons. Thus, the criteria were applied to 273 patients, while remainders were considered ineligible. Statistics and finals number of eligible candidates were compared to the inclusive database of 371 cardiac arrest ECPR candidates.
